# Supplementary material for: BdCV1-Encoded P3 Silencing Suppressor Identification and Its Roles in Botryosphaeria dothidea, Causing Pear Ring Rot Disease
Source: Cells. 2023 Sep 29;12(19):2386. doi: 10.3390/cells12192386 (PMC10571871; doi:10.3390/cells12192386)
Supplement: Supplementary file 1 [file cells-12-02386-s001.zip › supplementary figures.8.28.pptx]

## Slide 1
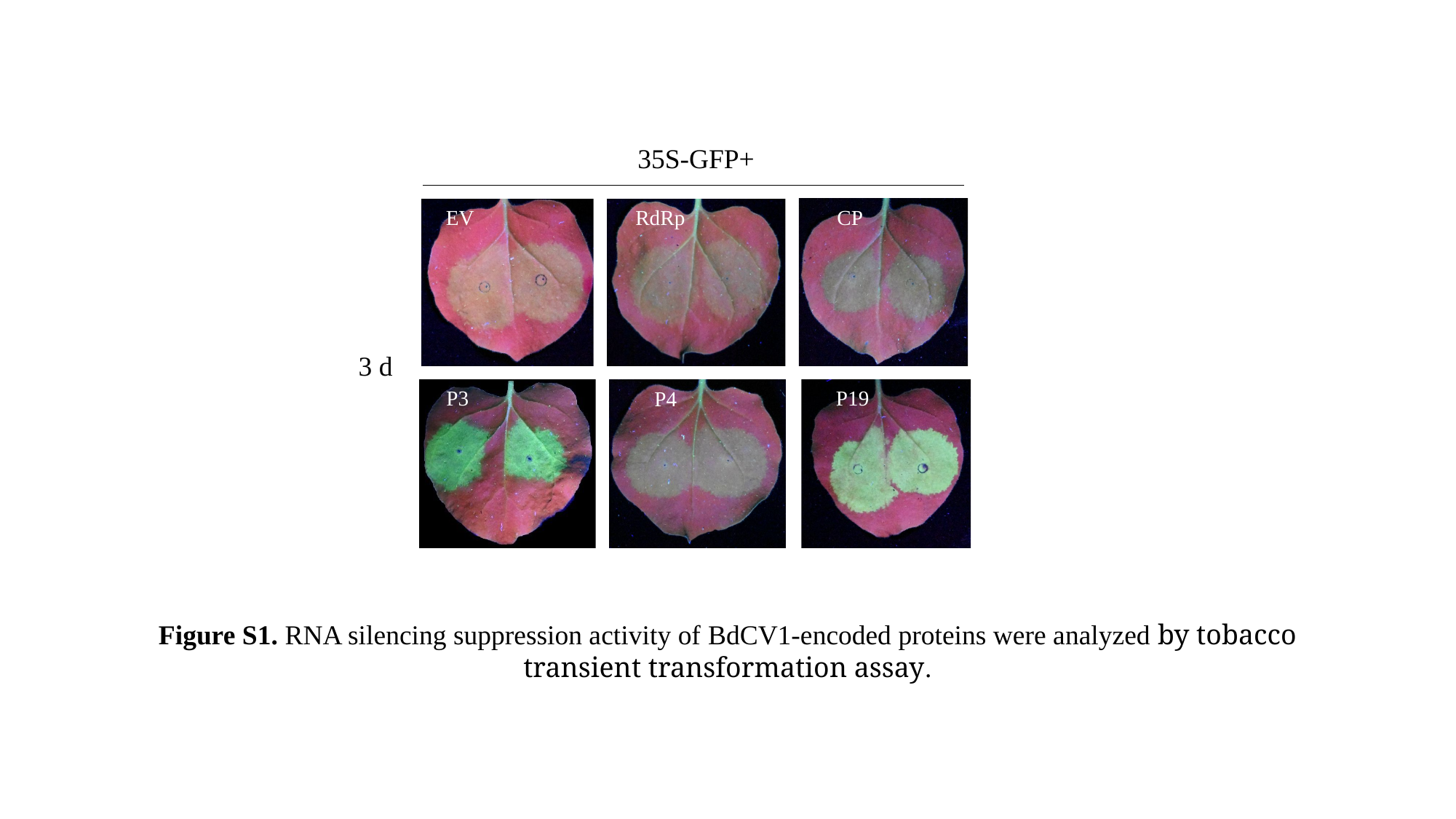

35S-GFP+
RdRp
EV
CP
P19
P4
P3
3 d
Figure S1. RNA silencing suppression activity of BdCV1-encoded proteins were analyzed by tobacco transient transformation assay.

## Slide 2
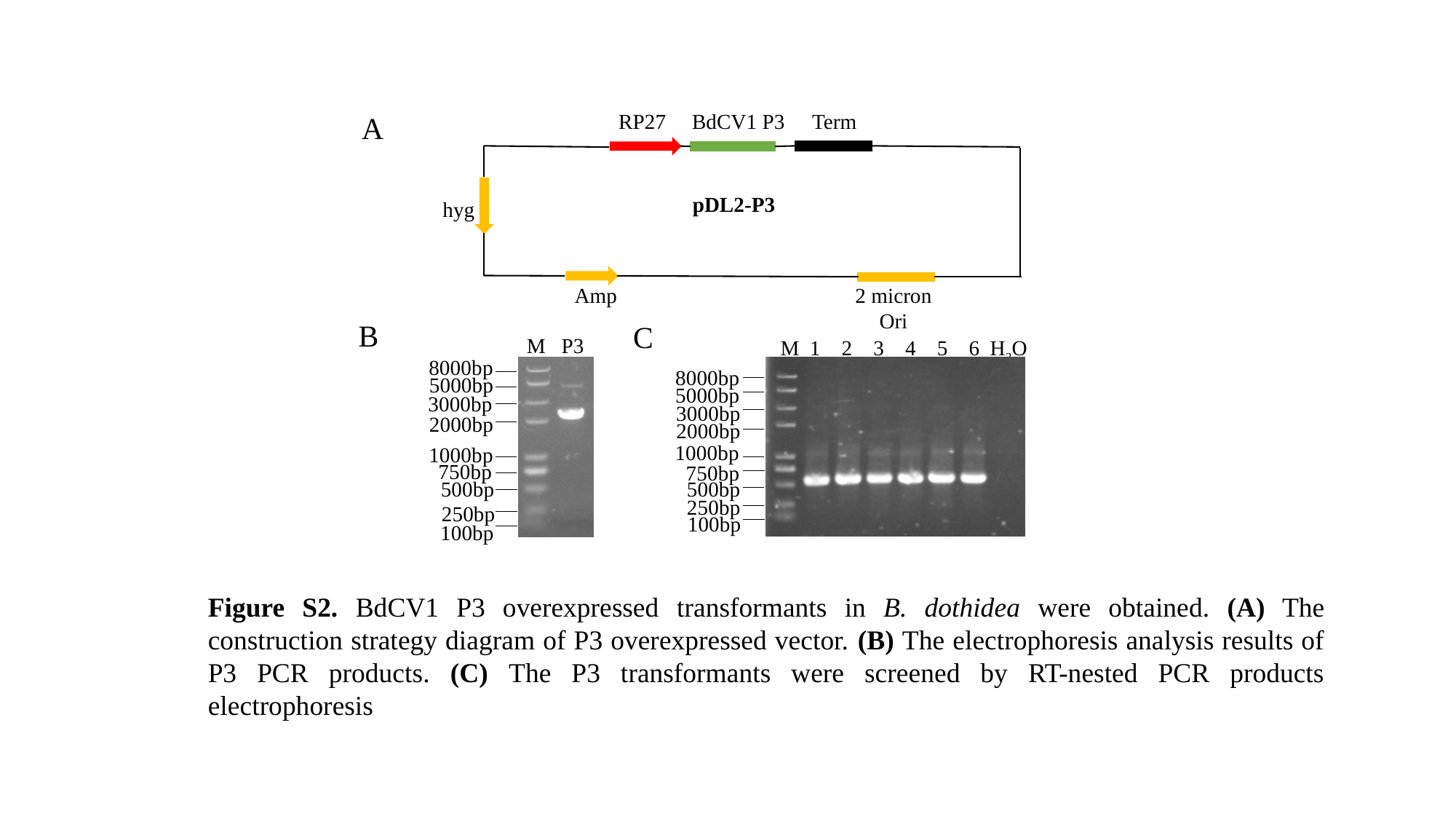

RP27
Term
BdCV1 P3
pDL2-P3
hyg
Amp
2 micron Ori
A
B
C
M P3
8000bp
5000bp
3000bp
2000bp
1000bp
750bp
500bp
250bp
100bp
 M 1 2 3 4 5 6 H2O
8000bp
5000bp
3000bp
2000bp
1000bp
750bp
500bp
250bp
100bp
Figure S2. BdCV1 P3 overexpressed transformants in B. dothidea were obtained. (A) The construction strategy diagram of P3 overexpressed vector. (B) The electrophoresis analysis results of P3 PCR products. (C) The P3 transformants were screened by RT-nested PCR products electrophoresis

## Slide 3
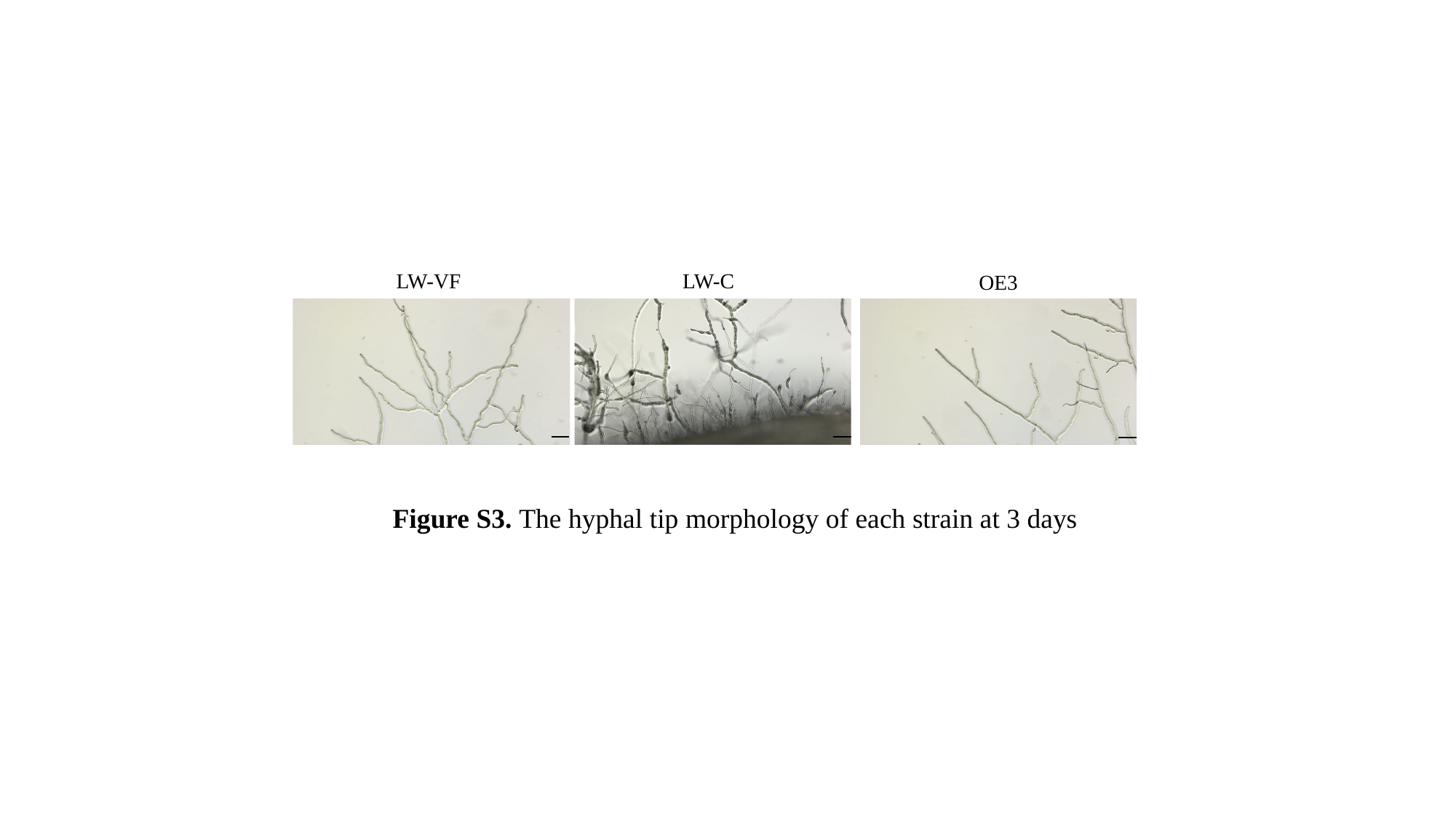

LW-VF
LW-C
OE3
Figure S3. The hyphal tip morphology of each strain at 3 days

## Slide 4
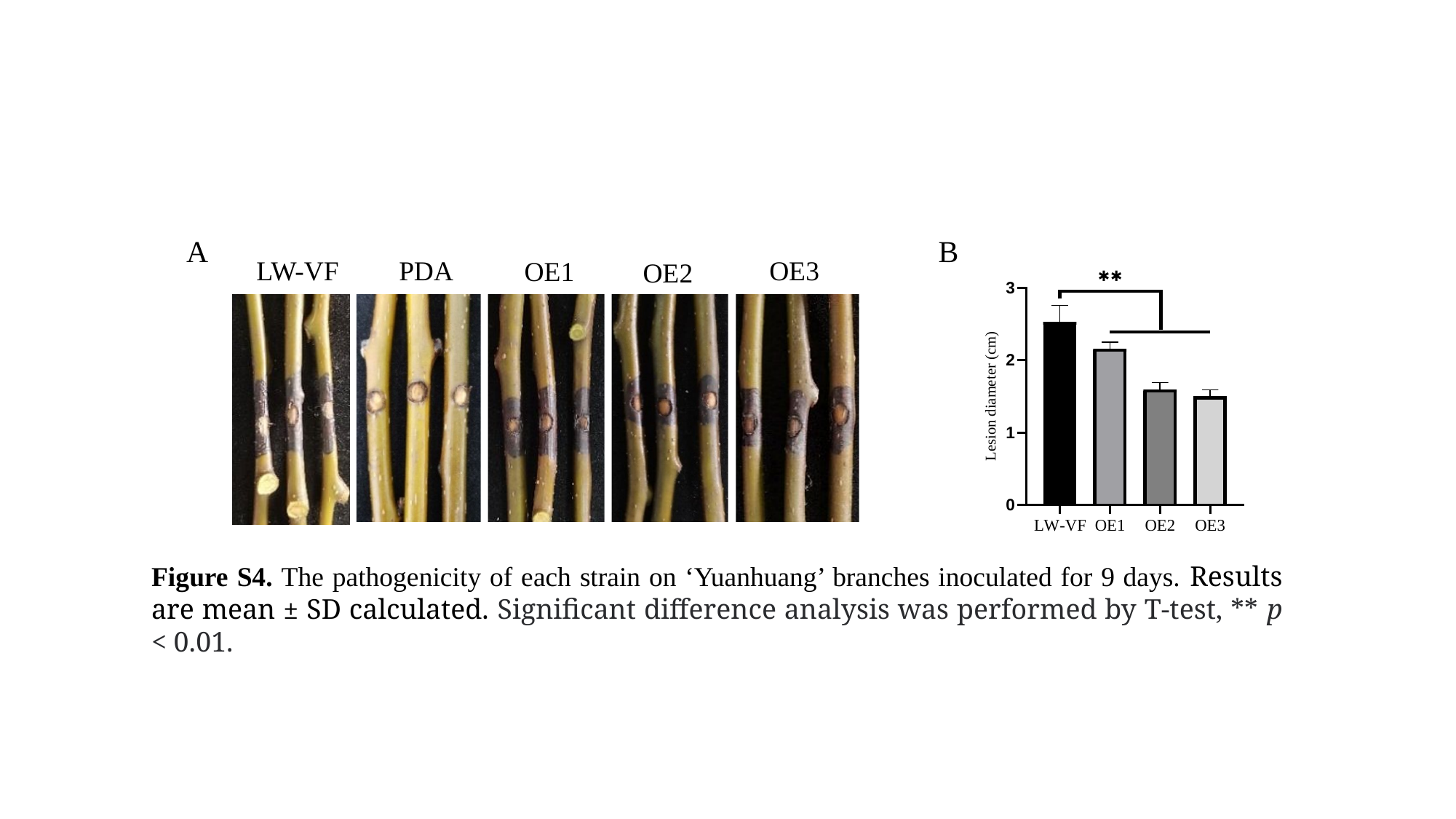

B
A
LW-VF
PDA
OE3
OE1
OE2
Figure S4. The pathogenicity of each strain on ‘Yuanhuang’ branches inoculated for 9 days. Results are mean ± SD calculated. Significant difference analysis was performed by T-test, ** p < 0.01.

## Slide 5
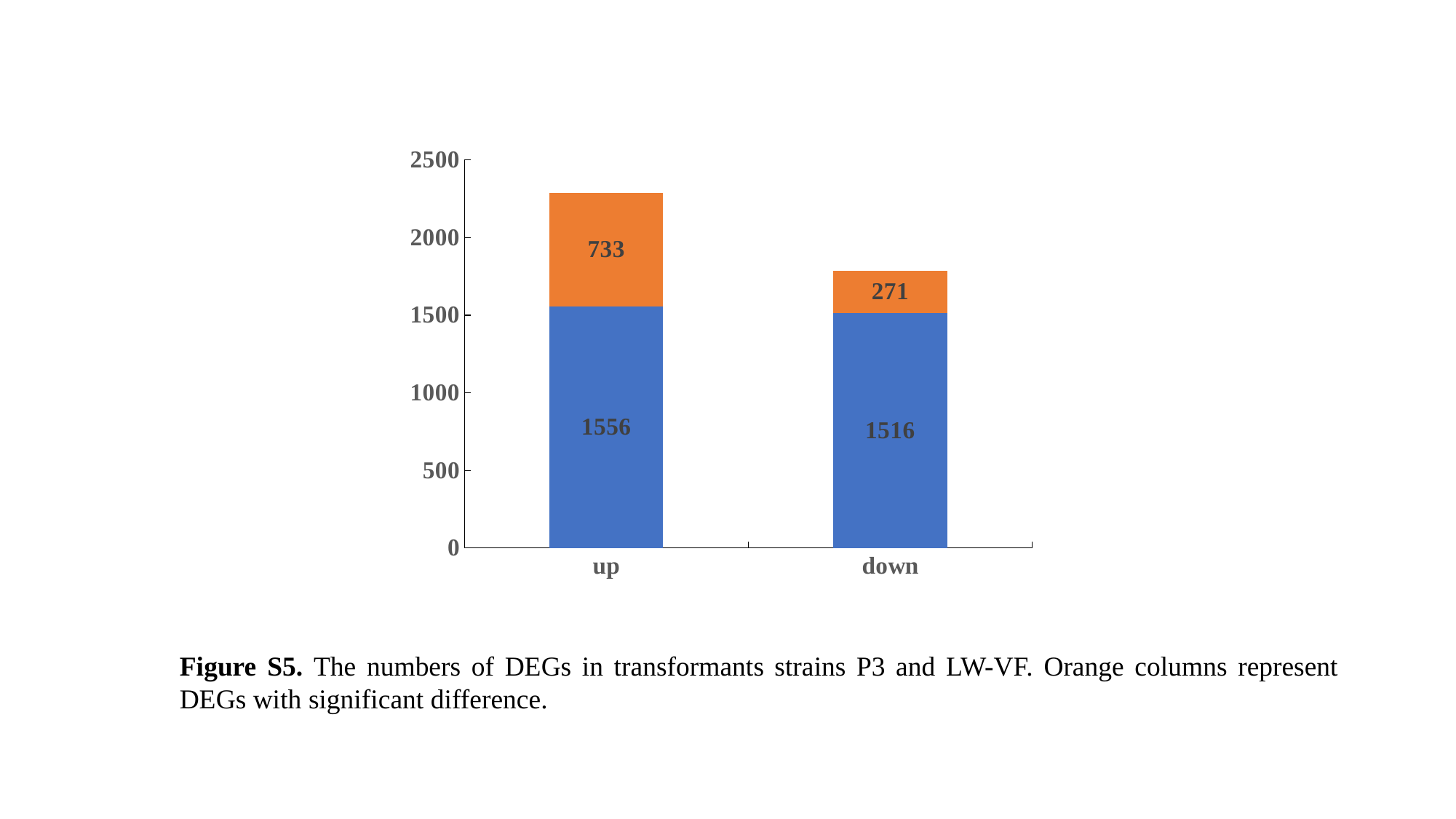

### Chart
| Category | | |
|---|---|---|
| up | 1556.0 | 733.0 |
| down | 1516.0 | 271.0 |Figure S5. The numbers of DEGs in transformants strains P3 and LW-VF. Orange columns represent DEGs with significant difference.

## Slide 6
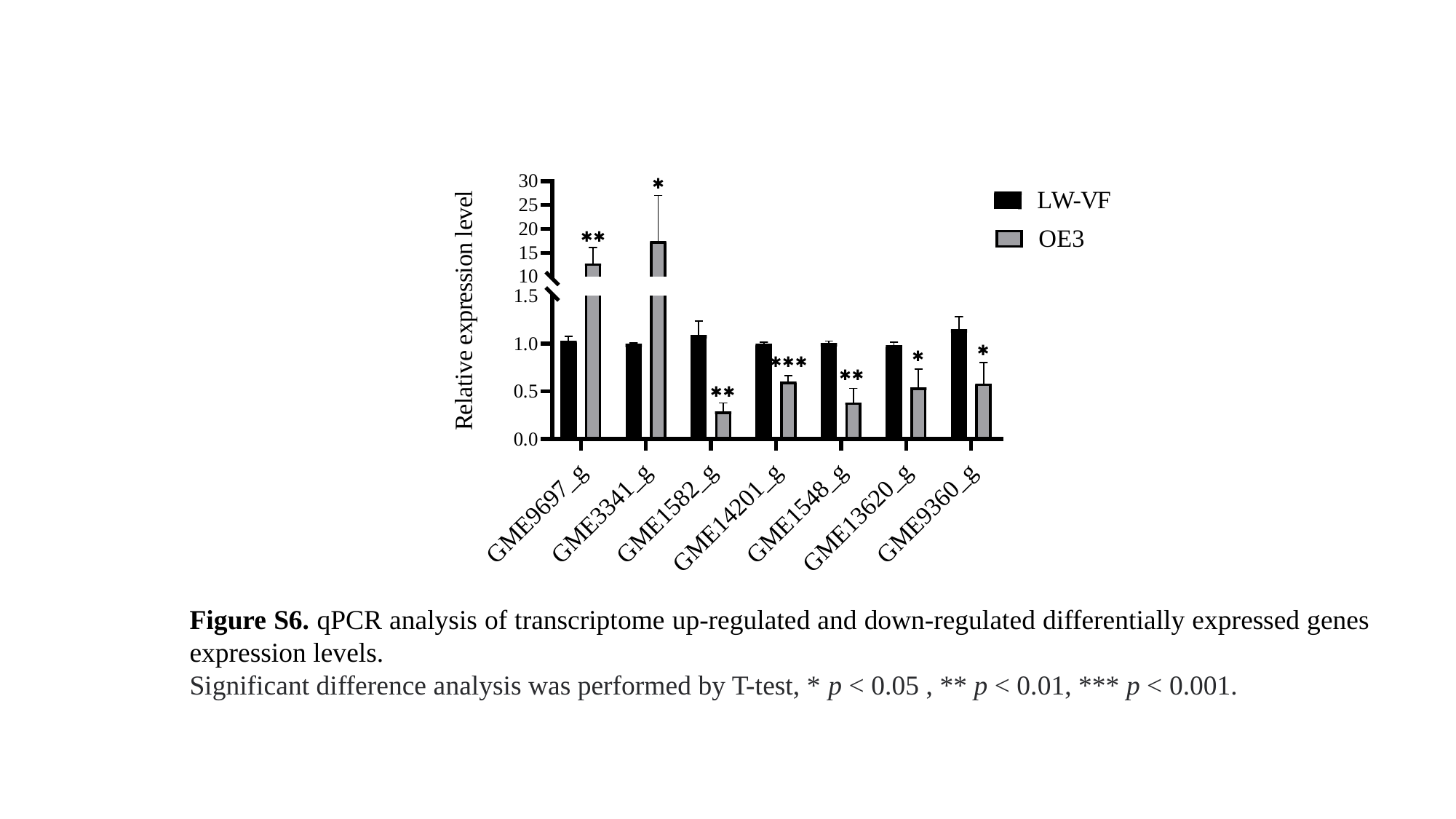

Figure S6. qPCR analysis of transcriptome up-regulated and down-regulated differentially expressed genes expression levels.
Significant difference analysis was performed by T-test, * p < 0.05 , ** p < 0.01, *** p < 0.001.

## Slide 7
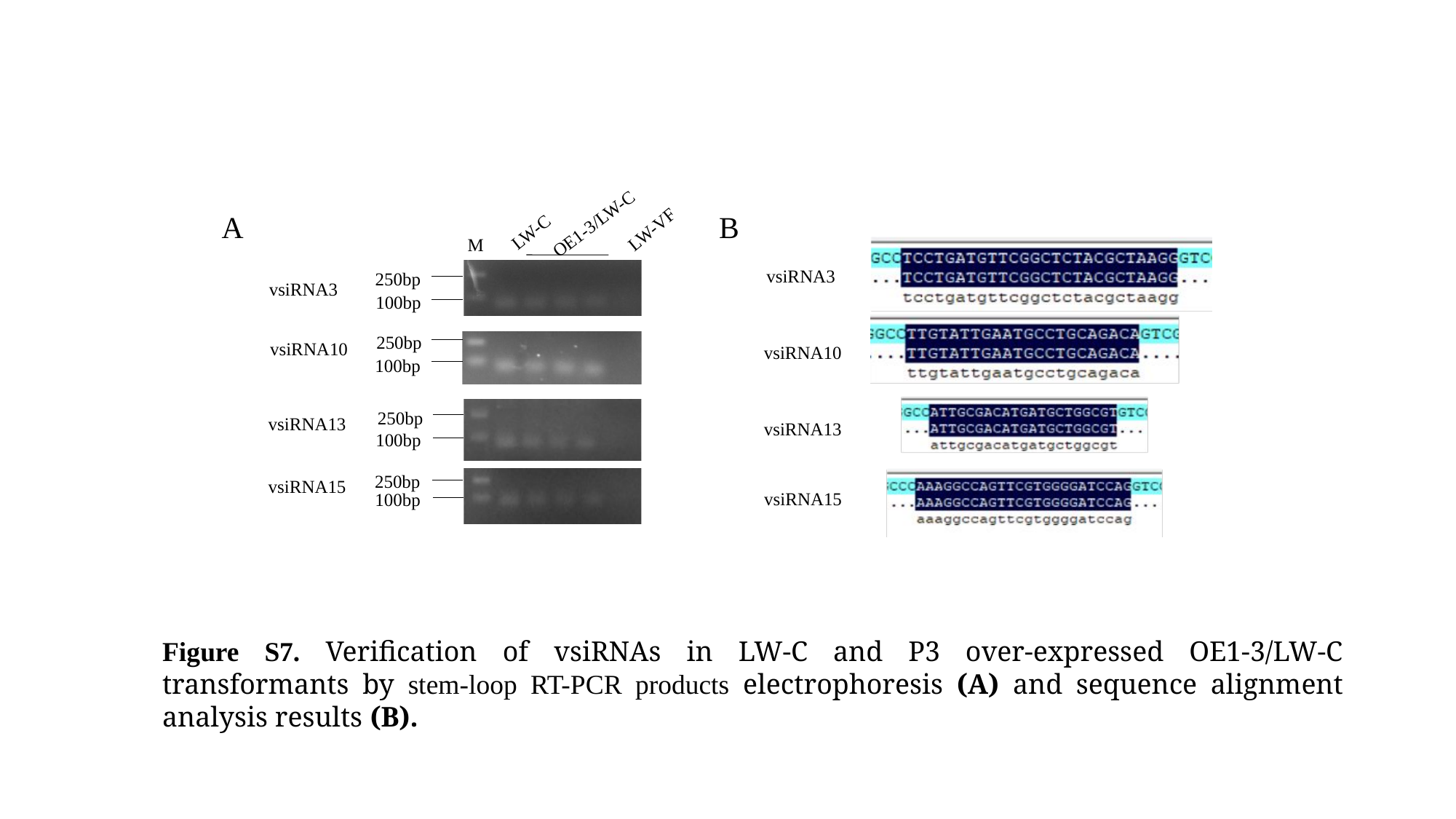

B
A
OE1-3/LW-C
LW-VF
LW-C
M
250bp
vsiRNA3
100bp
250bp
vsiRNA10
100bp
250bp
vsiRNA13
100bp
250bp
vsiRNA15
100bp
vsiRNA3
vsiRNA10
vsiRNA13
vsiRNA15
Figure S7. Verification of vsiRNAs in LW-C and P3 over-expressed OE1-3/LW-C transformants by stem-loop RT-PCR products electrophoresis (A) and sequence alignment analysis results (B).
