# Supplementary material for: BdCV1-Encoded P3 Silencing Suppressor Identification and Its Roles in Botryosphaeria dothidea, Causing Pear Ring Rot Disease
Source: Cells. 2023 Sep 29;12(19):2386. doi: 10.3390/cells12192386 (PMC10571871; doi:10.3390/cells12192386)
Supplement: Supplementary file 1 [file cells-12-02386-s001.zip › Supplementary tables.pdf]

Table S1 The primers information in this work

Primer name	Sequence（5'-3'）	Function
RdRp F	ATGTCAGCCACACTAGGTGCTT	Fragment amplification
RdRp R	TTAGTAGTACATGTAACC	
CP F	ATGACTTCCGCCGACAAC	
CP R	TCATGATGCCACTAACAC	
P3 F	ATGGCCTCCTACGAACAAC	
P3 R	TCACACGAGTTGGCGCAT	
P4 F	ATGAGTTTCACTCGTGACC	
P4 R	TTATGGCAATACACCACGC	
pCNF3-RdRp F	ACCATTTACGAACGATAGCATCTAGAATGT CAGCCACACTAGGTGC	Vector construction
pCNF3-RdRp R	CGGATCCACTAGTAGGCCTTCTAGAGTAG TACATGTAACCATCG	
pCNF3-CP F	AAAACTAGTATGACTTCCGCCGACAAC	
pCNF3-CP R	CGGGGTACCTGATGCCACTAACACTATCTC G	
pCNF3-P3 F	ACCATTTACGAACGATAGCATCTAGAATG GCCTCCTACGAACAAC	
pCNF3-P3 R	CGGATCCACTAGTAGGCCTTCTAGATCAC ACGAGTTGGCGCAT	
pCNF3-P4 F	TGCTCTAGAATGAGTTTCACTCGTGACCC	
pCNF3-P4 R	CGGGGTACCTGGCAATACACCACGCATG	
pDL2-P3 F	GTAGGAACCCAATCTTCAAAATGGCCTCC TACGAACAAC	
pDL2-P3 R	TGAATGTTGAGTGGAATGATTCACACGAG TTGGCGCAT	
P3-J F	GGCCAGTGGTAACGGCAA	
P3-J R	AAGCGGCCCAGTCCGGAGCA	
pDL2-mexp-JC F	CGTTGTCGCCTAACAGATC	
pDL2-mexp-JC R	GGGTATTGCCTTTGGACTT	
NbPR1 F	CATAACACAGCTCGTGCAGATGTA	RT-qPCR for gene expression level analysis of PRs
NbPR1 R	ACCTGGAGGATCATAGTTGCAAGAG	
NbPR2 F	CATAACCTTCCACTCTTA	
NbPR2 R	GATACAATAATCTCCACATT	
NbPR5 F	ATTCGGAGGACAACAATA	
NbPR5 R	GTGCTAGTAGGATCATCTT	
NbPR4 F	GAACAGTCCAGGCTCGCATT	
NbPR4 R	CCGTTACAGTCTCCAGTCTCA	
GAPDH F	GCAGTGAACGACCCATTTATCTC	
GAPDH R	AACCTTCTTGGCACCACCCT	
AGO1 F	CACAACAAAGCCCGTCTGGAG	

AGO1 R	GAATCAGAACCTTGCCCGACT	RT-qPCR for gene expression level analysis of RNAi components
Dicer2 F	GGCTGAGATGGTCGAGGAG	
Dicer2 R	CTTGCTGATGGCAGAGGGT	
BdActin F	CCGCTCCGTTTCTATGCTCT	
BdActin R	ACCCTCACCGACATACCAGT	
vsiRNA3 RT	GTCGTATCCAGTGCAGGGTCCGAGGTATTCGCACTGGATACGACCCTTAG	PCR fragment amplification and RT-qPCR analysis of vsiRNAs
vsiRNA3 F	GGCCTCCTGATGTTCGGCTCTA	
vsiRNA10 RT	GTCGTATCCAGTGCAGGGTCCGAGGTATTCGCACTGGATACGACTGTCTG	
vsiRNA10 F	GGCCTTGTATTGAATGCC	
vsiRNA13 RT	GTCGTATCCAGTGCAGGGTCCGAGGTATTCGCACTGGATACGACACGCCA	
vsiRNA13 F	GGCCATTGCGACATGAT	
vsiRNA15 RT	GTCGTATCCAGTGCAGGGTCCGAGGTATTCGCACTGGATACGACCTGGAT	
vsiRNA15 F	GCCCAAAGGCCAGTTCGTGG	
Stem loop R	GTGCAGGGTCCGAGGT	
GME9697 F	TACTTCCATGCCGCGCTA	RT-qPCR for mRNA expression level analysis of selected genes from transcriptome
GME9697 R	AACTCCAGCCGCGAAGTA	
GME3341 F	AGGACCAGCCAATTCCCA	
GME3341 R	ATCCAGCGTCCAGCAACC	
GME1582 F	GTCTCAGATGTCCTCGGC	
GME1582 R	ATGCGCGAGAATCCACAC	
GME14201 F	TCGATCCGTTGGCCGATATG	
GME14201 R	CTCGACTTCAGACGCGATCA	
GME1548 F	AACGTGGAGGGGATCGAG	
GME1548 F	TGCCTCTGGTACTGCCTG	
GME13602 F	GAGCCGGTCCAAAACCCATA	
GME13620 R	CCAGCTTGACACCGGATTCT	
GME9360 F	TTCCGGGATACCAGCCCTAA	
GME9360 R	CTGTTGGCGAATGTATGCGG	

Table S2 The phenotype of 35S-GFP + P3, 35S-GFP + P19 and 35S-GFP + EV infiltrated *N. benthamiana* line 16c leaves

Infiltration treatment	Infiltrated leaves	Necrotic leaves
35S-GFP+P3	35	35
35S-GFP+P19	17	1
35S-GFP+EV	13	0

Table S3 The quality statistical analysis reads filtered from raw reads of *B. dothdiea* by RNA-Seq

Sample	Total Raw reads (Mb)	Total Clean reads (Mb)	Total Clean Bases (Gb)	Clean reads Q20(%)	Clean reads Q30(%)	Clean reads Ratio(%)
LW-VF 1	43.69	42.44	6.37	98.25	93.22	97.14
LW-VF 2	43.69	42.45	6.37	98.59	94.59	97.16
OE3 1	43.69	42.73	6.41	98.43	93.98	97.8
OE3 2	43.69	42.34	6.35	98.49	94.22	96.91

Table S4 Sequencing information of vsiRNA

Name	Polar	Position	Sequence（5’-3’）	Length
vsiRNA3	+	2222-2247	TCCTGATGTTCGGCTCTACGCTAAGG	26 bp
vsiRNA10	-	77-98	TTGTATTGAATGCCTGCAGACA	22 bp
vsiRNA13	+	946-966	ATTGCGACATGATGCTGGCGT	21 bp
vsiRN15	+	1836-1859	AAAGGCCAGTTCGTGGGGATCCAG	24 bp
